# Research on Application of Meticulous Nursing Scheduling Management Based on Data-Driven Intelligent Optimization Technology

**DOI:** 10.1155/2022/3293806

**Published:** 2022-09-12

**Authors:** YanPing Zhai, Run Li, ZhiLi Yan

**Affiliations:** ^1^Internal Medicine Teaching and Research Section, Shanxi Bethune Hospital, Shanxi, Taiyuan 030032, China; ^2^School of Nursing, Shanxi University of Chinese Medicine, Shanxi, Taiyuan 030024, China

## Abstract

The management of nursing scheduling in healthcare facilities have faced new challenges during the COVID-19 pandemic. With the rapid development of big data and artificial intelligence technology, data-driven intelligent medical services are what we need to study nowadays. This paper not only proposes reasonable solutions in areas such as refined nursing scheduling by using these scientific technologies to quickly realize the allocation of human resources in hospitals. It also accelerates the development of hospital informatization construction through computer technology, establishing a scientific and intelligent medical platform that meets the needs of users. Aiming at the problem of nursing scheduling in medical service data research, this paper proposes a complete plan by analyzing the development of the medical platform at this stage. Firstly, established an intelligent medical service platform, and studied the medical management from the perspective of data. Then, analyze the intelligent medical platform data by utilizing optimized algorithms, through reasonable analysis under various constraints, to get the basic nursing scheduling plan that meets the needs of medical institutions. Finally, considering the actual situation of emergency medical treatment, the decision classification model is introduced under the basic scheme to further screen out the optimal management scheme of modern medical treatment.

## 1. Introduction

The sudden outbreak of COVID-19 has had a severe impact on the lives of all mankind. Hospitals are the institutions most severely affected by the epidemic, mainly due to the centralized distribution of patients, which leads to the unbalanced allocation of medical resources and indirectly leads to a significant increase in the prevention and control costs and operating costs of medical institutions. COVID-19 has not only had a significant impact on the global healthcare industry, but also made more and more people value healthcare more. In the post-epidemic era, innovation and progress in the medical industry have become the focus of social attention, and patients' medical thinking and habits will change dramatically. Hospitals need to face the challenges and speed up the information construction process of smart medical treatment [1]. In recent years, the problem of nurse scheduling has been concerned by many hospital administrators and related scholars [2]. A good scheduling is of great significance to the development of the unit. However, with the development of social information, manual scheduling has been obviously outdated, and nurses scheduling information is an inevitable development trend. Intelligent scheduling informatization based on big data is a multidisciplinary problem of nursing, management, operations and computer. Researchers need to have a more comprehensive understanding of this problem and solve it through performance-guided design, by improving market share and competitiveness, and then achieve sustainable development of hospitals.

At present, the world population is still increasing, and the development of medical care is in a difficult stage. At the same time, the demand for medical personnel brought by the increase in the number of patients has not been well met, and there are also problems such as waste of health expenses, resulting in inadequate allocation of medical and health human resources [3]. What's more, hospital medical service efficiency is low. In the case of insufficient allocation of health human resources, there is still some idle personnel, wasting a large number of medical and health resources. Medical care in most countries is still in the low information stage of manual scheduling. Most hospital scheduling is done manually by the head nurse through experience. Such scheduling has many disadvantages. First of all, manual scheduling is inefficient and wastes a lot of time and resources. And with the development of modern hospitals, the number of nurses in the department and their needs are gradually increasing, and it will become more and more difficult to manually schedule shifts. Secondly, if multiple nurses put forward preference requirements, the head nurse is prone to be influenced by subjective factors in the schedule and cannot meet the requirements of each nurse with fairness and justice, which may cause dissatisfaction among nurses and also the conflicts between nurses and hospital management [4].

**Figure 1:**
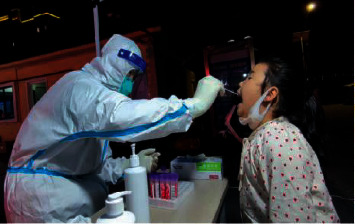
Nurses' working pictures from Chongqing daily news report.

Therefore, the whole society should pay attention to how to effectively control hospital medical costs under the background of limited medical service resources. It is very important to improve the overall service quality of a hospital to ensure the service efficiency of medical and health personnel by rational allocation of nurses [5]. Considering that nurses serve patients in hospitals, the negative emotions of patients with diseases may also indirectly affect nurses' emotions, resulting in certain psychological pressure [6]. In addition to the influence brought by the special nature of nurses' work, there is a wrong concept of valuing medical care over nursing in the society, which is mainly influenced by factors including traditional culture. These adverse impacts on nurses' life and work, at the same time, affect patients' satisfaction with medical services, which may cause tension between nurses and patients [7, 8]. Then it will lead to greater uncertainty in the allocation of hospital human resources. When nursing work is heavy, additional temporary nurses need to be hired to meet the demand for medical and health services, which increases hospital costs to a certain extent. As shown in Figure 1, there are nurses doing nucleic acid tests for people during the COVID-19 pandemic.

On the one hand, although the overall strength of the global medical system has been greatly improved, the medical service system in most countries still faces great work pressure. Due to the imbalance between supply and demand of medical staff and patients, in order to better serve the people, it is necessary to optimize and reform the human resource management of hospitals [9]. Maximize the potential of hospital medical staff. Human resource management plays a very important role in both public and private hospitals. If the human resource management is inadequate and the professional strength of the staff cannot be fully brought into play, the waste of talents will be caused. On the other hand, In the long-term application of the traditional nursing scheduling mode, its shortcomings are gradually revealed. The traditional medical model is platform management, and nurses usually only perform their own duties [10]. Especially for young nurses, their lack of nursing experience and poor emergency response ability will increase the risk of adverse nursing events [11]. More importantly, nurses' psychological pressure will lead to lower work enthusiasm, thus forming a vicious circle. The rationality of the traditional scheduling method for nursing staff is not high. Sometimes, during the consultation period, the nurses will be busy and affect the observation of the patient's condition and health education, increasing the contradiction between the doctor and the patient.

The way to solve the above problems is to arrange nurses' working shifts reasonably, which can not only ensure the efficient completion of hospital nursing work, but also ensure that nurses can fulfill other social roles. As countries pay more and more attention to the medical and health field, hospitals should not only ensure the quality of service, but also try to meet the individual needs of medical staff from their perspective, by improving job satisfaction, and service efficiency to a certain extent [12]. Through the rational application of big data technology, artificial intelligence technology and the medical industry, the efficiency of human resource management has been effectively improved. For example, in the comprehensive evaluation of medical staff, it is no longer necessary for staff to collect all kinds of resources. Through the application of big data technology in the computer system, it can quickly call up the medical staff's attendance resources, daily work data, certificate data, diagnostic records, occupational assessment data, mental health data, etc [13]. A comprehensive and detailed personal career report is also available for human resource management. Meanwhile, the big data technology will also use the data comparison of job requirements to provide human resource managers with a reference job adjustment plan. Through daily work and post adjustment programs provided by big data technology, human resources can better carry out the allocation of medical human resources, ensuring the effective implementation of the work of various departments and departments, and promoting the overall operation quality and service effect of the hospital [14].

To sum up, COVID-19 certainly has had a significant impact on the medical industry. Medical service with Internet plus initiative will promote the trend of direct connection between doctors and patients. Hospitals need to accelerate the construction of smart medical informatization, improve the ability of refined operation and management, and follow the trend to achieve sustainable development of hospitals. For industrial upgrading and industry development, the traditional medical industry has encountered many challenges and difficulties in labor management. For example, focus on the needs of different sub-scenarios in the medical industry, help the needs of refined labor management in the medical industry, and digitally empower enterprises to reduce costs and increase efficiency.

COVID-19 has accelerated the trend of direct access between doctors and patients. Hospitals should follow the trend, take precautions to build a smart hospital, by catching the express train named medical service with Internet plus initiative, to realize the sustainable development of the hospital. Accelerate the construction of the Internet hospital website, simulating the hospital entity, to increase the hospital brand weight in the virtual, and then improve the competitiveness with third-party platforms. Increase the information construction of hospitals, to meet the needs of patients' online medical consultation and inquiry to a greater extent, and improve the hospital's influence and market occupancy rate. Create online registration and hospital treatment channels to provide more convenient and fast medical services. Organization is fundamental. The hospital should set up a team, an operation department or a health service department, which is specially responsible for the operation and management of the Internet hospital, or entrust third-party platforms to manage and operate it. It is necessary to give full play to the commanding role of performance appraisal to guide and accelerate the advancement of medical service with Internet plus initiative. For those who provide services and attract hospital diagnosis and treatment through the hospital Websites, increase their performance incentives, fully revitalize their time, increase their treatment, and mobilize their enthusiasm, to achieve coordinated development with hospitals.

## 2. Related Studies

Hospital human resource management directly affects the overall service quality and effect of the hospital [15]. In order to provide patients with more efficient and safe medical services, it is necessary to carry out some reforms and innovations in hospital human resource management [16]. The intelligent scheduling system combines the business digital model to automatically calculate the demand of each position and generate the scheduling plan intelligently, convenient and efficient scheduling operation, preset a variety of scheduling rules. Different types of employees adopt different scheduling strategies to fully meet the multi-business needs of the medical industry. Enable the scheduling is real-time and transparent, that is, employees can view it at any time, effectively improving employee satisfaction and controlling labor costs. Nursing scheduling must conform to certain rules, if the arrangement of too few people, can not ensure the normal operation of the ward and the patient's nursing needs. If there are too many people in the queue, the overflow of manpower will waste resources and increase the operating cost of the hospital. A good nursing scheduling system should be able to meet the needs of nurses' personal preference, nursing staff energy level, nurses' workload, shift balance, basic manpower requirements of each shift and other multi-dimensional requirements.

Western scholars have studied the scheduling problem of nurses earlier and put forward various methods in the assays. The nurses' scheduling model is mainly based on single objective model or changing multi-objective model into single objective problem model by weighting method. The research objectives mainly include reducing hospital salary costs, minimizing violation of hospital regulations, and maximizing the satisfaction of nurses' preferences. In addition, sometimes hospitals also consider how to reduce the number of nurses and reduce the overtime hours of nurses. Burke et al. [17] proposed heuristic sorting and variable neighborhood hybrid algorithm to solve nurse scheduling problems in reality by using weighted cost function as evaluation index. Sarkar et al. [18] proposed a heuristic framework with new features and a multi-parameter cost function based on variable waiting time. Finally, genetic algorithm toolbox was used to prove the feasibility of the framework and cost function. Mutingi and Mbohwa [19] proposed a simulation algorithm based on the concept of biological evolution to apply to nurse scheduling, which took into account user choice, user expectation and expert intuition and experience of decision makers.

The solution methods of nurse scheduling mainly include mathematical programming method and heuristic method. Most of the early studies adopted mathematical programming solution method. Musa and Saxena established mathematical programming model for several nurses and solved the problem using objective programming method [20]. Maenhout and Vanhoucke established a nurse scheduling model aiming at the penalty value of violation of nurses' assigned shifts, adopted the weighted sum method to transform the multi-objective problem into a single objective problem, and adopted the branch and bound method to solve the problem, and discussed the advantages and disadvantages of different branches and pruning methods in different scenarios [21].

However, the advantage of the accurate algorithm is that it can find the exact optimal solution of the problem, but the disadvantage is that for large-scale problems, the algorithm has high time complexity, and sometimes cannot be solved. The scale and high computational complexity of nurse scheduling problem determine that it is not suitable to be solved by an accurate algorithm. Legrain et al. [22] proposed a heuristic method based on local search, which is flexible, simple and easy to implement, and better than CPLEX solution. Topaloglu [23] proposed a multi-objective planning model of nurse scheduling with 10 hard constraints and 9 soft constraints. The sequential method and weighting method were used to transform the multi-objective function into a single penalty function for solving. Parr and Thompson [24] proposed to use neighborhood search to solve the nurse scheduling problem using weighted cost function, and conducted a comparative experiment between sawing method and simulated annealing noise method, which proved that noise method could get a better scheduling scheme.

In recent years, more and more scholars use mixed mathematical methods to solve the problem of nurse scheduling. This is because the accurate algorithm consumes too much time to solve the multi-constraint and large-scale nurse scheduling problem and even cannot find the optimal solution. There are some defects in the heuristic algorithm, and there is no perfect scheme. Tassopoulos et al. proposed a two-stage variable domain search algorithm, which improved 48 instances in the benchmark problem of nurse competition and achieved better results [25]. Jaumard et al. promoted and extended the previous nurse scheduling model, proposed the 0–1 target nurse scheduling model based on the shortest path problem with resource constraints, and solved the model with column generation algorithm [26]. In recent years, there are also many studies on combinatorial algorithms, which can achieve better solution results. The optimization objectives proposed by Bard and Pumomo included minimizing mobile nurses and maximizing nurse satisfaction, establishing a multi-objective model, solving it by using column generation and integer programming, and completing an example verification in an American hospital [27]. Awadallah et al. proposed a hybrid algorithm using harmony search and mountain climbing algorithm, which can enhance the search ability of the algorithm and is suitable for solving a variety of nurse scheduling problems [28].

With the expanding influence of computer technology on all walks of life, there are more and more cross applications of science and technology and medical treatment. Abdennadher and Schlenkef supported semi-automatic generation of scheduling tables by imitating some thinking modes of human beings based on the idea of constraint programming [29, 30]. Cheng et al. [31] introduced and implemented a constraint-based nurse scheduling system by using a redundant modeling method. Hofe developed a nurse scheduling system based on hierarchical constraint satisfaction method on the basis of existing studies, and assays lists the practical application of this system [32]. In order to quickly get high-quality scheduling schedules, Muslija proposed an intelligent backtracking algorithm consisting of four steps and applied it into commercial software [33]. Also based on the variable neighborhood search algorithm, Burke et al. [34] proposed a hybrid heuristic sorting and variable neighborhood search algorithm (HVNS). Firstly, the algorithm uses heuristic method to generate the initial solution, and then optimizes the initial solution with variable neighborhood search algorithm. Finally, in order to jump out of the local optimal, the algorithm uses a heuristic restart mechanism to disturb the local optimal solution. Rahimian et al. [35] proposed a hybrid integer programming and variable neighborhood search algorithm. The algorithm first uses a greedy heuristic algorithm to generate the initial solution, and then optimizes the solution using a variable neighborhood descent algorithm.

The study predicts the patient-related indicators, and then get the nurse allocation situation in a period of time in the future with the consideration of the total nursing time required by the patient, the effective nursing working time of nurses and the ratio of nurses to other medical staff, so as to formulate a reasonable method of nurse allocation according to the actual demand of the hospital. In the article [36], Valouxis and Housos adopted an approximate integer linear programming model to generate the initial solution of the nurse scheduling problem, and then adopted the local domain search method to optimize the initial solution. Nikola Todorovic proposed a colony optimization algorithm to solve the nurse scheduling problem in assay [37]. The algorithm simulates the foraging behavior of bees, including the process of scheduling and local search. Santos et al. proposed an integer programming method to solve the nurse scheduling problem in assay [38]. The algorithm starts to build the model of the problem and gives the integer programming formula of the problem. By referring to this formula, the algorithm gives a method to improve the performance of the IP solver.

Nursing scheduling is an important part of improving the efficiency of daily nursing management and rationally allocating hospital human resources [39]. The electronic nursing schedule can reduce the workload of the head nurse, facilitate the information exchange between different levels, and facilitate the nursing department to understand and manage the whole hospital manpower arrangement. In view of the low efficiency of paper scheduling and the diverse demands of different wards for scheduling, this paper designed a data-driven intelligent nursing scheduling management system, which fully considered the diversity of wards and the complexity of medical scheduling. This article aims to solve the hugely different needs of different wards for the setting of shifts and scheduling rules, and try to meet the diverse needs of each ward as much as possible.

## 3. Smart Medicine Based on Data

### 3.1. Data-Driven Medical Cloud Platform

The rapid development of Internet technology, promotes the continuous evolution of society towards informatization, networking and digitization. The focus of the Internet era has gradually shifted from the “Internet of Information,” with cloud computing, big data, and mobile Internet as the underlying technologies, to the “Internet of Value,” with block-chain, cryptography, and distributed consensus as the underlying technologies. A new round of technological revolution with block-chain as the underlying technology is coming. In recent years, block-chain technology and the field of smart medicine have been booming. Industry and academia have begun to try to combine these two, using block-chain's features of de-centralization, high fault tolerance, imtamability, traceability, anonymity and many others to solve the problems of data storage and privacy protection in the field of smart medicine. Then, realize storage and secure sharing of medical data among multiple entities. Medical image data continues to increase, and the amount of data stored, archived and invoked continues to rise; A large amount of data is stored and the interoperability is weak. Therefore, it is difficult to establish a unified shared resource management platform. The limitation of distance and talent makes primary medical care powerless. Doctor-patient information is sensitive, and information storage and sharing are difficult to be synchronized. This series of industry demand, all need to be supported by information technology, from a single system.

Medical information is about everyone's health information, and user data privacy is about ethics and regulation. For a long time, information and data in the medical industry have been in a closed environment. The information construction needs to get through the data between different platforms, and the data privacy, data security and other issues involved have been hindering the information construction of the medical industry. Due to the particularity of the medical industry, there are many personalized needs, and the needs of each department are different. Many hospital departments have the problem of independent division, and each department has a set of business, resulting in difficult data access and integration. With the development of medical equipment, more and more large-capacity archival data need to be preserved. These data grow very fast, and a large number of data need to be generated every day. At the same time, these data often need to be preserved for a long time. As the filing system of each hospital is not open, the medical data storage of the hospital is confronted with great challenges. Slow medical treatment has always been a pain point in the medical industry. How to solve this problem with the help of the digital process of the medical industry is the top priority in the digital transformation. With the emergence of big data analysis and AI technology, IT technology can help boost the diagnosis and treatment process, which can greatly save users' time to see a doctor. However, the performance of traditional IT architecture is difficult to meet the needs of real-time analysis.

**Figure 2:**
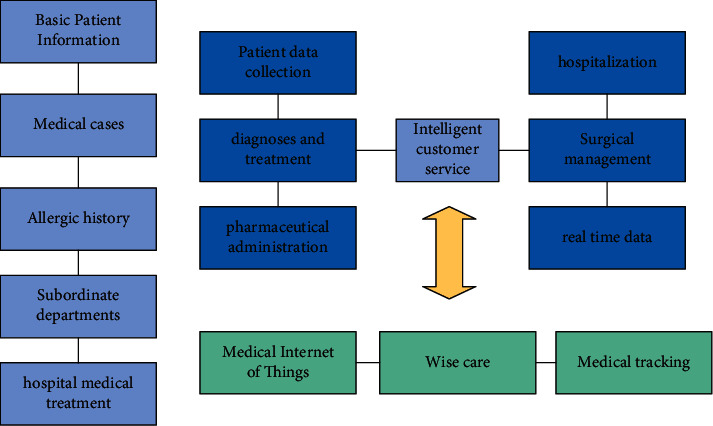
Data-driven framework of intelligent medical system.

In the development of hospital human resource management, the sharing mechanism of hospital medical information can be built based on big data technology. Through the sharing and construction of medical information, the connection between various departments and departments can be effectively improved and the overall improvement of hospital service quality can be promoted. When the information of the hospital's human resources management is incorrect, it cannot promote the efficient development of related work. In order to solve this problem well, it is necessary to build a hospital's internal information management system, improve the circulation efficiency of hospital's internal information, and ensure that human resource management can play its corresponding work value. The framework of the smart medical platform based on the form of shared data is shown in Figure 2 in this paper. Data of multiple types and different departments are integrated and intelligent analysis is integrated to ensure the best results can be obtained through sharing.

### 3.2. Particle Swarm Optimization Algorithm Based on Medical Treatment

Particle swarm algorithm is also called particle swarm optimization algorithm (PSO). Simulate the behavior of birds randomly searching for food. In particle swarm optimization, the potential solution to every optimization problem is like a bird in the search space, called a particle. All particles have a fitness value determined by the optimized function, and each particle has a velocity that determines the direction and distance they “fly.” The algorithm has the following advantages: simple and easy to operate, fast convergence speed, few setting parameters. And the general limitation condition is that when the number of steps or the corresponding accuracy is reached, the particle will stop moving. Particle swarm optimization algorithm, as a kind of algorithm to find the optimal value, mainly relies on the iterative formula, which is updated as follows:

(1) $$\begin{array}{c} V_{i\;d}=wV_{i\;d}+C_{1}\text{random}\left(0,1\right)\left(P_{i\;d}-X_{i\;d}\right)+C_{2}\text{random}\left(0,1\right)\left(P_{g\;d}-X_{i\;d}\right),\\ X_{i\;d}=X_{i\;d}+V_{i\;d}, \end{array}$$

where *w* is the inertia factor and ***C*** is the acceleration constant. *P*_*i* *d*_ represents the *d*-th dimension of the individual extremum of the *i*-th variable, and *P*_*g* *d*_ represents the *d*-th dimension of the global optimal solution.

Particle swarm optimization (PSO) initializes as a group of random particles (random solutions) and then iterates to find the optimal solution. In each iteration, the particle updates itself by tracking two extreme values: the first is the optimal solution found by the particle itself, which is called the individual extreme; The second is the optimal solution found so far for the whole population, which is called the global extremum. Or instead of using the entire population, you can use a fraction of it as a neighbor, called a local extremum. Combined with the actual hospital situation, it is necessary to consider the long-term effect of nurses on duty. When on duty, because the night shift work hours are long and reversed with the normal working hours, nurses generally do not want to work night shift, and the number of night nurses is small, so the night nurses are selected first, and the other nurses work day shift. First of all, according to the principle of voluntary, nurses who show willingness to work night shift will be given priority, and nurses who show reluctance to work night shift will be given priority under the same conditions. Secondly, according to the principle of fairness, nurses who worked less night shifts in a certain period of time (such as one year) should be considered first, and then nurses who did not work night shifts in the previous period should be considered, so as to avoid excessive fatigue caused by nurses working night shifts for too long.

**Figure 3:**
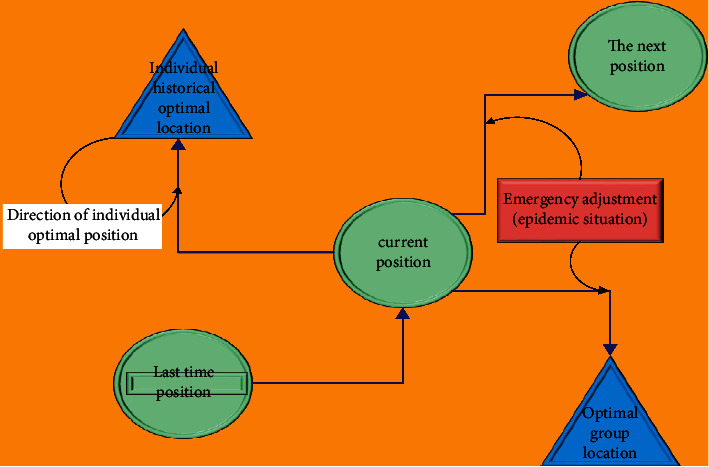
Update graph of nurse scheduling based on particle swarm optimization.

The update process of particle swarm optimization algorithm based on nurse scheduling is shown in Figure 3. The neighborhood topology of PSO includes two kinds: one takes all the individuals in the population as the neighborhood of the particle; the other takes only some individuals in the population as the neighborhood of the particle. Neighborhood topology determines the optimal location of community history. Thus, PSO is divided into global PSO and local PSO. The global particle swarm algorithm includes the historical optimal value of the particle itself and the global optimal value of the particle population (the optimal value is determined by all particles).

As a swarm intelligence algorithm, particle swarm optimization has an obvious advantage that it can be encoded by real numbers instead of binary codes, and is easy to operate. Update mechanism of particle swarm optimization algorithm is only the global optimal solution to the information of other particles. And the algorithm does not need too much need to adjust the parameters. At the same time, the whole process with the current global optimal solution is updated iterations, also can quickly converge to global optimal location nearby, showing the fast convergence of the particle swarm optimization algorithm, the effectiveness and robustness, etc. At the same time, particle swarm optimization algorithm can achieve fast convergence to the final result, but it is easy to produce local optimal results. How to balance local optimization ability and global search ability is worth paying attention to, otherwise it is difficult to achieve good convergence effect.

Due to the impact of COVID-19, nurses have to pay more attention to epidemic prevention and control on the basis of their original duty, so the smart scheduling system should also take these factors into account. First, we divided the area into medium-high risk area or low-risk area, and then analyzed the data of medical duty on this basis. The first point is that the nurse clearly states the rest date and whether the nurse can work on the first day. (If the nurse worked the night shift on the last day of the previous cycle, the nurse may not work on the first day of the new cycle). The third point is to determine the continuous work of the nurse at the end of the previous week. These data are used to calculate the continuous work of the nurse at the beginning of the new cycle. Fourth: determine the amount of work done by the nurse in the week when the new and old cycles are connected, and determine the workload of the nurse in the first week of the new cycle. The third and fourth items in the analysis information connect the old and new cycles, which is conducive to the hospital's fair and equitable management of nurses in the long term. According to the actual medical situation, the patient flow of the hospital was analyzed and predicted. We conducted preliminary detection according to the machine learning algorithm, which was convenient for the subsequent specific analysis of nurses on duty.

On the basis of intelligent forecasting, nurses were divided into day shift nurses and night nurse nurse in both groups. If the nurse scheduling for three shifts, chronic shift nurses can be divided into three groups. Thus when a nurse to work day shift, the night is no longer need to consider, so can reduce a lot of calculation, also meeting the requirements of the hard constraints directly, making the problem more simple. In addition, in the three-shift system, the working hours of each shift are generally similar, and the nurses' preference is not obvious. The classification should first consider the types of shifts that the nurses explicitly indicate to work or not to work, and then the nurses who are insufficient will be randomly assigned. In previous studies when nursing was a rapid shift, all nurses might work all shift types and there was no need to group nurses.

### 3.3. Medical Decision Classification Based on Big Data

Medical big data has a long history. There are written reports of clinical cases in ancient times. And with this, Islamic doctors in the Middle Ages further developed case records for teaching purposes. The forerunners of modern case records first appeared in Paris and Berlin in the early nineteenth century. Subsequently, clinical case records in the United States were developed in some teaching hospitals. In the twentieth century, clinical case data records were developed for direct use in patient care in hospital and outpatient Settings. There are many kinds of classification methods in data mining, such as decision tree method, FC nearest neighbor method, support vector machine, clustering algorithm, Logistic regression method, artificial neural network algorithm and so on. Among them, decision tree method is widely used in many problems because of its advantages of generating understandable rules, relatively small amount of calculation, processing continuous variables and classified variables, and clearly displaying which variables are more important.

Decision tree is a basic classification and regression method. The model of decision tree is a tree structure, which is usually composed of nodes and directed edges. There are two types of nodes: intermediate nodes and leaves. The intermediate node usually represents a certain feature or attribute in the decision-making process, while the leaf node represents a classification or regression result. When leaf nodes represent classification results, such decision tree is also called classification tree. When leaf nodes represent regression results, a prediction real value is usually given. Such decision trees are also called regression trees. When using decision tree for classification or regression, a certain feature of the instance is tested starting from the root node. According to the test results, the instance is assigned to its corresponding child nodes, and then the process is repeated until the leaf node is reached. Finally, the instance is assigned to the corresponding category of the leaf node or the predicted value of regression.

XGBoost is an abbreviation for eXtreme Gradient Boosting, which has solved many real-world decision problems in recent years. XGBoost's excellent performance (both effectiveness and speed) made it the top solution in the screen data science competition for a long time, and it is still the model of choice for many big machine learning solutions. XGBoost is very excellent in parallel computing efficiency, missing value processing, control overfitting and prediction generalization ability. The base classifiers supported by XGBoost include decision trees and linear models. To prevent overfitting, XGBoost sets tree-based complexity as the regular term:

(2) $$\begin{array}{c} \Omega \left(f\right)=\gamma T+\frac{1}{2}\lambda {\left\|w\right\|}^{2}, \end{array}$$

where, ***T*** is the number of leaf nodes of tree *f*, *w* is the vector formed by the output regression values of all leaf nodes. ‖*w*‖^2^ is the square of the *L*2 norm (modulus length) of this vector, and *γ*, *λ* is the hyperparameter. The objective function is as follows:

(3) $$\begin{array}{c} obj=\sum_{i=1}^{N}\left[g_{i}f_{m}\left(x_{i}\right)+\frac{1}{2}h_{i}f_{m}^{2}\left(x_{i}\right)\right]+\gamma T+\frac{1}{2}\lambda \sum_{j=1}^{T}w_{j}^{2}. \end{array}$$

XGBoost also provides an approximate version of the above greed criterion, in short, using feature quantiles as segmentation candidates. In this way, the set of candidate points is reduced from traversal between the whole sample to traversal between several quantiles. Specifically, there are two strategies for selecting feature quantiles: global and local. Global is selected from the eigenvalues of all samples, and it only needs to be performed once before root node splitting. Local is selected from the eigenvalues of the samples contained in the nodes to be split, which should be carried out before each node is split. In general, since a global can only be partitioned once, it needs to be partitioned at a finer granularity.

Medical data is relatively special, so XGBoost optimization is a good fit for medical decision making. In order to speed up the selection of optimal segmentation points, XGBoost divides the eigenvalues into buckets according to the density distribution of eigenvalues, and uses the boundary value of the bucket as the candidate of the splitting points. Therefore, before training, the eigenvalues should be pre-sorted to find out the candidate cutting points, and then saved as a block structure, which is repeatedly used in subsequent iterations, greatly reducing the amount of calculation. In the process of node splitting, the gain of each feature needs to be calculated, and the feature with the largest gain is finally selected to split.

Comprehensively consider the characteristics of nurses on duty, the gain calculation of each feature can be carried out by using multi-thread. That is, multi-thread parallel method is used to find the optimal segmentation point on different feature attributes, which can also play a role in preventing over-fitting. More importantly, the calculated splitting gain does not contain samples of missing values when searching for splitting points during training. In terms of logical implementation, in order to ensure completeness, samples missing the eigenvalue will be assigned to the left leaf node and the right leaf node respectively. After calculation of gain, the direction with large gain will be selected to classify samples containing the missing value. In the prediction stage, if there is no missing value in the training set but missing value in the test set, the default direction of the branch should be specified for the missing value (or the value that the specified value does not appear), and the missing value will be automatically divided into this branch during the prediction.

## 4. Experimental Results and Analysis

### 4.1. Experimental Setup

In the basic experiment phase, we chose the public data set INRC-2010, which consists of the test set provided by the 2010 contest, and mainly contains three types of questions: Sprint, Medium and Long. Each type of problem consists of three use cases: Early use cases, Late use cases, and Hidden instances. Sprint problems, Medium problems, and Long problems increase in size. The size of the problem for the early, Late, and Hidden instances of the same type increased. Through the above data to verify all the algorithms involved in this chapter, we compare and analyze the results of the algorithm in this paper with the best results of existing algorithms to verify the effectiveness of the algorithm in this paper. In order to verify the model more accurately, this paper will also simulate actual medical data for verification, the basic hardware requirements of the experiment are shown in Table 1.

Nurse scheduling is a NP-hard problem, which is small in scale but complicated. Python language is suitable for the implementation of nurse scheduling algorithm. In addition, Python is a good language for implementing this algorithm because of the excellent performance of machine learning algorithms. Python has the advantages of numpy, Matplotlib, SciKit-Learn, PANDAS, ipython and other tools in scientific computing. Pandas has the unrivable advantage in handling medium data. It has become a major analysis tool in data analysis. Of course, Python also has powerful programming capabilities, which are different from *R* or MATLAB. Python has some very powerful data analysis capabilities, and it can also be used for crawler, game writing, and automatic operation and maintenance, which are widely used in these fields. These advantages make it possible for one technology to solve all business service problems, which fully demonstrates Python's ability to merge businesses. Using Python can greatly improve the efficiency of data analysis.

### 4.2. Basic Time Analysis of the Model

Given the special nature of the healthcare industry, the value of time is Paramount. In order to verify the performance of the algorithm effectively, we analyze and compare the experimental results of the ANS algorithm and the algorithm in this paper from multiple perspectives. And then analyze and compare the advantages and disadvantages of the results obtained by the two algorithms and the length of the algorithm's running time (experiment S data Sprint, *M* said data Medium, BKS is known about the best results of the use case, Fb is algorithm each nurse scheduling problem cases to get the best solution, Fa is obtain the average solution algorithm to solve the use case, Ta is the average time for the algorithm to solve each use case).

**Figure 4:**
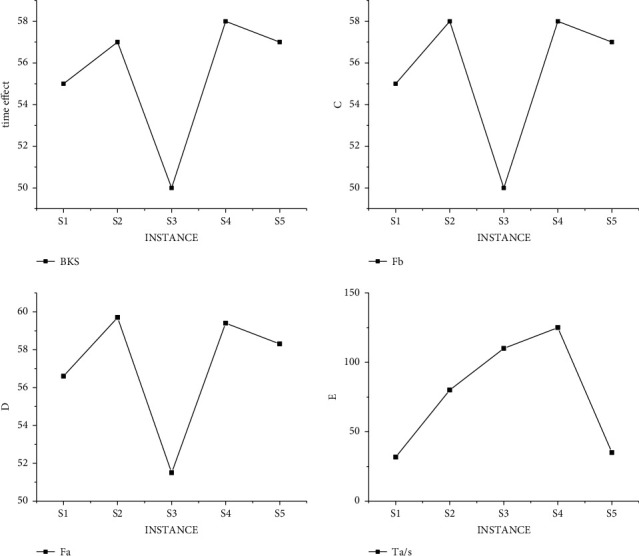
Experimental results of traditional algorithm in data S.

Through traditional analysis and verification of examples in the dataset, the experimental results are obtained as shown in Figure 4. It can be clearly seen from Figure 4 that the variation rules of graph Fb, the best analysis result of the data obtained by the algorithm, and Graph Fa, the average result of the algorithm, are almost identical. The variations in the three plots are the same, which means that the optimal scheduling choices are the same. And in the analysis of individual data, the scheduling results are better than the fixed standard, which proves that the algorithm can greatly improve the efficiency of hospital nurses on duty. However, to our regret, not all of the results were better than the norm, and some experimental results did not meet the norm.not all of the results were better than the norm, and some experimental results did not meet the norm.

**Figure 5:**
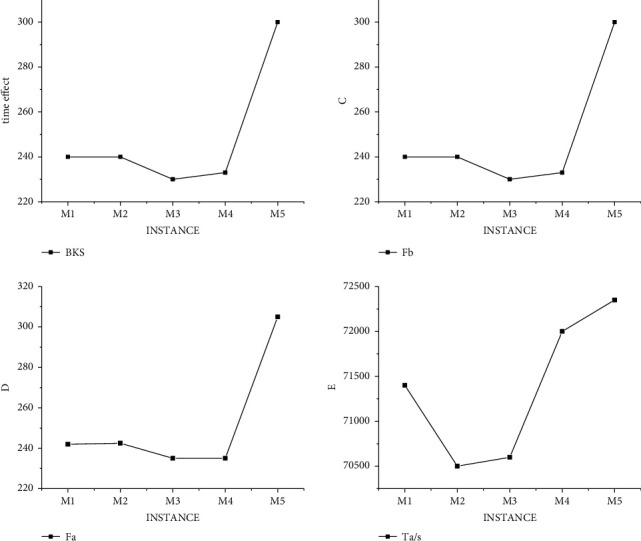
Experimental results of traditional algorithm in data M.

In order to exclude the error of the same type of data, we conducted the same experiment under data M, and the experimental results as shown in Figure 5 met the standard, but some data did not get the best value. That is, some data are not arranged in the best way, which may lead to the decline of hospital service evaluation in practical application.

**Figure 6:**
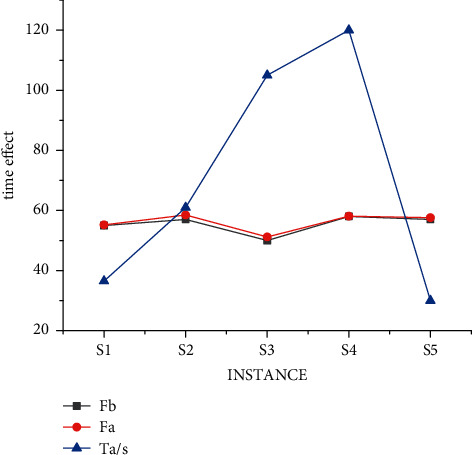
Results of mixed decision model under S data.

In order to address the shortcomings of the traditional algorithm, our improved hybrid decision model was also verified under the data S and M. And Figure 6 shows the experimental results of the hybrid model under the data S. By comparing Figures 4 and6, we can find that the number of optimal choices of the hybrid model is more than that of the traditional method, and it is easier for our algorithm to obtain the scheduling results of hospital demands based on the actual life.

**Figure 7:**
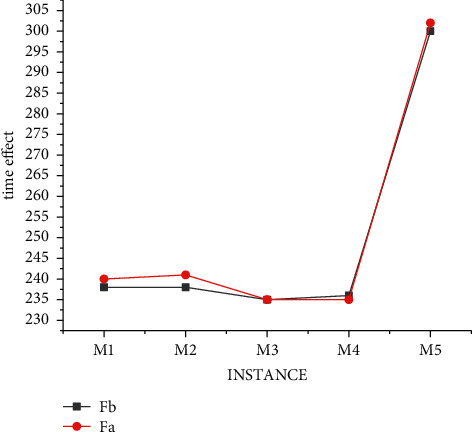
Results of mixed decision model under M data.

In order to further verify the validity of the model, the experimental results of the mixed model under M data are shown in Figure 7. By comparing Figures 5 and 7, we can find that the number of optimal choices of the hybrid model is still more than that of the traditional method. More importantly, the time of obtaining the standard value of the algorithm in this paper is significantly shorter than that of the traditional algorithm, indicating that the data processing efficiency of the algorithm is greatly improved.

**Figure 8:**
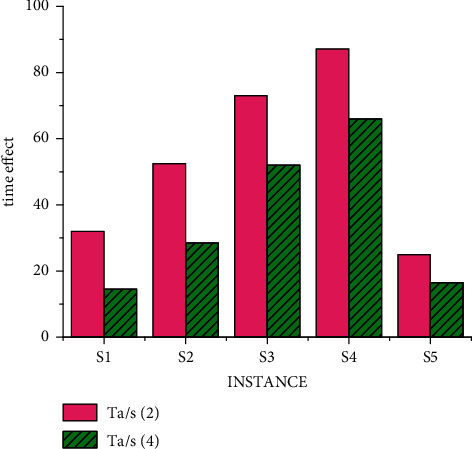
Time result of mixed decision model under S data.

The research on nurse scheduling problem belongs to the problem of selecting the optimal solution. This kind of algorithm research can further improve the processing efficiency through multi-threading (the time calculation of a scheduling problem is carried out under conventional splicing operation). The results of different threads of the mixed decision model under data S are shown in Figure 8. We obtained the experiment with small data through a large number of experiments, and the experimental result is that when solving each use case, all parameters of the two algorithms are the same, that is, the ending condition of the algorithm is the same. In this way, the best solution and the average solution are not calculated separately according to the different number of threads. But under the condition of three threads, a unified value can better prove the objectivity of the experimental results. It can be seen from Figure 8 that the results of four threads are significantly better than those of two threads, but the progress is not linear due to data changes, which also conforms to the actual law. That is, there are individual emergencies in medical scheduling that we have to take into account.

**Figure 9:**
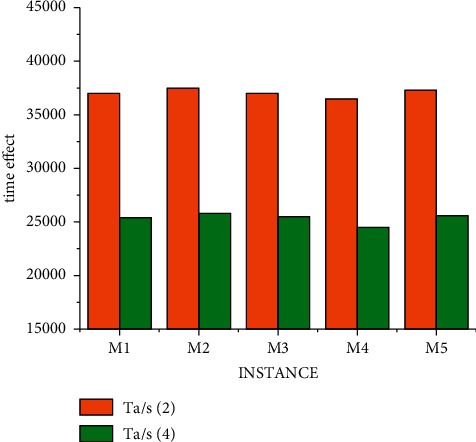
Time result of mixed decision model under M data.

By comparing the differences between Figures 5, 6 and 9, it is obvious that under the condition of conventional splicing, the running time of the improved hybrid algorithm is significantly reduced compared with that of the traditional algorithm. It shows that compared with random initialization methods, heuristic initialization methods generate high-quality solutions, which can reduce the number of iterations to a certain extent. And the experimental results show that the optimal solution of the hybrid algorithm is at least as good as that of the traditional algorithm, and the average solution is better than that of the conventional algorithm, and its time is shorter than that of the traditional algorithm. In addition, by comprehensive comparison of experimental results under data S and M, the best solution obtained by the algorithm in this paper in use cases is the same as the known best solution, and the obtained solution is even better than the known best solution. And it can be seen that with the increase in the number of threads, the average time for solving use cases is getting shorter. This shows that the time performance of the improved algorithm is better than that of the traditional algorithm.

## 5. Conclusion

With the development of artificial intelligence, human beings have entered the era of intelligent data. How to give full play to the maximum value of data is a common problem for all scientific researchers. Medical data application is one of the promising research fields. Hospital is a very special unit, involving a lot of professional departments and related departments. Under the impact of the global COVID-19 pandemic, the work of various departments and departments has become more difficult, so it is necessary to improve the rational allocation of existing resources in hospitals. Through the combination of optimization algorithm and decision algorithm, this paper proposes a scientific nursing scheduling scheme to ensure that professional medical staff can give full play to their strengths and provide medical services for patients. Only by closely combining computer technology with dependent services, can we realize the reform and innovation of hospital human resource management, ensuring the effective implementation of various human resource assignment work and enhancing the comprehensive strength of staff. In the future, more science and technology will be introduced into the medical industry. With the development of hardware technology, the talent gap of hospital departments will be filled, and the overall service potential of the hospital will be fully exerted.

## Figures and Tables

**Table 1:** Experimental environment configuration table.

Configuration	Parameters
Operating system	Windows10 (64)
CPU	Inter(R)Xeon(R)4116
Internal storage	128G
GPU	Tesla T4
Programming language	Python3.6
The text tool	BERT

## Data Availability

The experimental data used to support the findings of this study are available from the corresponding author upon request.
